# Dr. Angle’s Reply to Dr. Talbot

**Published:** 1899-08

**Authors:** Edward H. Angle

**Affiliations:** St. Louis, Mo.


### Domestic Correspondence.

### DR. ANGLE'S REPLY TO DR. TALBOT.

To the Editor:

Sir,—In the May number of the International Dental Journal we again hear from Dr. Talbot in regard to the operation of double resection of the lower maxilla. Evidently the doctor winces under Dr. Lukens's criticism (International Dental Journal, February, 1899) of his intermeddling and childish and obsolete methods of attempting to establish priority, but instead of answering and, if possible, refuting the criticisms of Dr. Lukens, he passes him by and directs his attention at me, although it is difficult to determine his object, or what points he really makes.

In his remarks he is rambling, but reiterates that the suggestion of this operation was not original with me, but with the late Dr. W. W. Allport, and that Dr. Allport mentioned it to him more than twenty-five years ago (by the way, Dr. Talbot's usual and favorite epoch when attempting to establish priority), but of this I have grave doubts for reason which I shall give later.

He further says that he (Talbot), with Drs. Allport, Brainard, and Gunn (all dead), was of the opinion that the operation was not indicated, from the fact that the pulps in the lower anterior teeth *might be* destroyed. "I have, therefore," says he, "refused to attempt the operation, although I have had a number of patients on whom such an operation might be performed with *éclat.*"

Now, if it is true that these men really did discuss this operation from twenty-five years to four decades ago (although we have not the least evidence of it, other than Dr. Talbot's word), and were intimidated from performing it through fear of the possible death of a few tooth-pulps (which fear has been proven groundless), permitting this possible disadvantage to outweigh the great advantages of changing a practically useless dental apparatus to one of real usefulness and normal function through the re-establishing of correct occlusion of the teeth, as well as changing the facial appearance from that of a repulsive deformity to certainly marked and pleasing improvement, if not real beauty, they must have been bold and eminent surgeons indeed. But we cannot see how the world has been benefited through their connection with the operation any more than if these men had never lived, or what right they have to any mention in connection with it, especially Dr. Talbot, who was sceptical and critical even after the operation was successfully performed, as shown in his first criticism in the INTERNATIONAL for October, 1898, although in his last article under discussion, after finding that the operation was a success, he manifests a most radical change of heart and has suddenly become enthusiastic over the advantages and possibilities of the operation.

The reader should also remember in connection with this question that Dr. Talbot has written some two works on the treatment of dental irregularities (both, however, now practically obsolete, I believe), in which some considerable attention is given to the treatment of the deformity under consideration. He has also, as is well known, written voluminously for the dental journals upon the subject, all of which have been profusely illustrated, as well as elaborated by tables of statistics (the reliability of which, however, has been questioned). Yet throughout all these writings there is not the least hint or slightest suggestion that he knew anything about the proposed operation. All who know him well will agree that this would hardly have been the case had the matter weighed upon his mind for four decades.

Now the facts with regard to my connection with the operation are these: Several years ago I became convinced, after seeing a number of cases of protrusion of the lower maxilla, that no devices or means known to the science of orthodontia would ever be successful in treating the pronounced cases, especially near or after maturity, and the thought occurred to me that we might successfully shorten the jaw by taking out sections on either side.

Naturally the first grave consideration was the difficulty of securing immovable apposition of the remaining segments of the bone, yet this seemed to me not difficult, for I at that time had several cases of fracture of the inferior maxilla which I had no trouble in immovably supporting, and I reasoned that the difficulties to be overcome in the proposed operation ought to be at least no greater than in cases of comminuted fracture, for with the former all the advantages of modern aseptic surgery could be invoked, while with the latter, ofttimes the most unfavorable conditions, accompanied by other lesions, must be met with.

Not long after this I discussed the operation with Professors Moore and Hunter (all living), of the Medical Department of the Minnesota State University, both well-known surgeons of Minneapolis, and still in successful practice there.

Soon after this, or January 18, 1893, in an illustrated lecture which I gave before the Minneapolis Dental Society on the "History of Treatment of Fractures of the Maxillæ," I described the proposed operation.

Since that time I have also described the operation and its possibilities before each of my classes in the American College of Dental Surgery and Dental Department of the Northwestern University, Chicago, and also in the Marion-Sims Medical College, Dental Department, St. Louis.

In another paper on "Treatment of Fractures of the Maxilla," given in December, 1895, before the St. Louis Dental Society, I also described the operation and its possibilities, which was discussed by Drs. McMillan, Fuller, Bartlett, Fletcher, Whipple, and others (all still very much alive).

During all these years I was anxious to find a suitable patient who was willing to have the operation performed. Once in Minneapolis I was consulted by a Mr. Cook, a medical student of the Minnesota State University, who readily comprehended the difficulties and possibilities, and was willing and anxious to have the operation performed, and Professor Moore and myself had nearly completed the preliminary arrangements. It was, however, prevented by vacations and change of my residence, much to the regret of both Professor Moore and myself, as the more we thought about the operation the more we became convinced it would be a success.

The illustrations used by me in the *Dental Cosmos* of August, 1898, in describing the plan of securing fixation in these cases, to which Dr. Talbot refers, were made by the S. S. White Dental Manufacturing Company more than one year previous to the operation performed by Drs. Tupper and Blair, assisted by Dr. Whipple, and were intended, as stated, to illustrate a description of the proposed operation in my forthcoming book.

There is another bit of history in connection with this operation which it now seems necessary to disclose since Dr. Talbot's last unkindly insinuations.

I knew the late Dr. Allport. He was my friend. He occasionally visited his son, Dr. Allport, who lived in Minneapolis. He always paid me several friendly calls during these visits, at which time we frequently discussed questions relating to dentistry, and especially the one which has been for several years with me most prominent,—orthodontia. Always, when I had any new ideas, I would discuss them with him, and on one of these visits I described to him this operation. He at first did not seem to understand the operation I was describing, and said, "Why, that is the same as Hullihen's operation." I said, "No, it is very different. This involves removal of a complete section of bone and alveolar process on each side, while Hullihen's consisted in shortening the arch of the alveolar process after first removing V-shaped sections from the alveolus alone." He then quickly grasped the difference and seemed to think it might be successful, but he did not evince the least evidence of ever having heard of the operation before, much less having discussed it with Dr. Talbot.

Now, in conclusion, had I not repeatedly advocated the operation, in all probability the first would not yet have been performed, and Dr. Talbot, still intimidated by the possible loss of a few tooth-pulps, would be sitting in his office viewing patients on whom he might have (but did not) performed such an operation with *éclat*.

EDWARD H. ANGLE.

ST. LOUIS, MO., May 17, 1899.

